# Supplementary material for: SpliceV: analysis and publication quality printing of linear and circular RNA splicing, expression and regulation
Source: BMC Bioinformatics. 2019 May 8;20:231. doi: 10.1186/s12859-019-2865-7 (PMC6507234; doi:10.1186/s12859-019-2865-7)
Supplement: Supplementary file 1 — Command line parameters for SpliceV. (PDF 251 kb) [file 12859_2019_2865_MOESM1_ESM.pdf]

Supplemental File 1

Usage:

splicev -b [one or more bam files] -gtf [one gtf file] -g [gene to plot]

all arguments:

-h, --help show help message

-b, --bam Path to bam file(s)

-t, --transcript Name of transcript to plot (must match "transcript_id" field of gtf file)

-g, --gene Name of gene to plot (overrides "-t" flag). Will plot the longest transcript derived from that gene

-gtf Path to gtf file

-bsj Path to backsplice junction bed-formatted files (listed in the same order as the input bam files)

-sj Path to canonical splice junction bed-formatted files (listed in the same order as the input bam files)

-stranded If strand-specific sequencing, indicate 'forward' if upstream reads are forward strand, otherwise indicate 'reverse' (True-Seq is 'reverse')

-is, --intron-scale The factor by which intron white space should be reduced

-c, --color Exon color. Hex colors (i.e. "\#4286f4". For hex, an escape "\" must precede the argument), RGB (i.e. 211,19,23) or names (i.e. "red")

-f, --filter Filter out splice junctions and circles that have fewer than this number of counts

-n, --normalize Normalize coverage between samples

-rc, --reduce_canonical Factor by which to reduce canonical curves

-rbs, --reduce_backsplice Factor by which to reduce backsplice curves

-ro, --repress_open Do not automatically open plot in browser

-en, --exon_numbering Label exons

-rnabp List of RNA binding proteins to plot.

-rnabpc Colors to use for RNABPs

-fa Path to fasta file

-format Output image format {SVG,PDF,PNG,JPG,TIFF}

-alu Path to Alu bed file
