# Supplementary material for: SpliceV: analysis and publication quality printing of linear and circular RNA splicing, expression and regulation
Source: BMC Bioinformatics. 2019 May 8;20:231. doi: 10.1186/s12859-019-2865-7 (PMC6507234; doi:10.1186/s12859-019-2865-7)
Supplement: Supplementary file 2 — An example pipeline to generate the required files for SpliceV analysis. (PDF 148 kb) [file 12859_2019_2865_MOESM2_ESM.pdf]

Supplemental File 2

Figure 1B

* gencode.v29.basic.annotation.gtf file obtained from
ftp://ftp.ebi.ac.uk/pub/databases/gencode/Gencode_human/release_29/gencode.v29.basic.annotation.gtf.gz

Download the file then run the following command:

```
gunzip gencode.v29.basic.annotation.gtf.gz
```

* GRCh38.primary_assembly.genome.fa file obtained from
ftp://ftp.ebi.ac.uk/pub/databases/gencode/Gencode_human/release_29/GRCh38.primary_assembly.genome.fa.gz

Download the file then run the following commands:

```
gunzip GRCh38.primary_assembly.genome.fa.gz
```

* STAR (v2.5.2a) and bowtie2 (v2.3.3) genome indices need to be generated prior to running this pipeline. More information can be found here:

- http://bowtie-bio.sourceforge.net/bowtie2/manual.shtml
- https://github.com/alexdobin/STAR/blob/master/doc/STARmanual.pdf

* Paired end FASTQ files can be obtained from the NCBI Sequence Read Archive (SRR7474078)

1. Generate **coverage** and **canonical splice junction** files:

```
STAR \
--genomeDir GRCh38 \
--runThreadN 14 \
--readFilesIn SRR7474078_1.fastq SRR7474078_2.fastq \
--outSAMtype BAM SortedByCoordinate \
--outFileNamePrefix SRR7474078.

# Convert canonical splice junction file to SpliceV compatible format
python SpliceV/bin/star_sj_convert SRR7474078.SJ.out.tab
```

2. Generate **back-splice junction** file with the find_circ pipeline (https://github.com/marvin-jens/find_circ):

```
bowtie2 \
-p 16 \
--very-sensitive \
--phred33 \
--score-min=C,-15,0 \
-x GRCh38 \
--reorder \
--mm \
-q \
-1 SRR7474078_1.fastq \
-2 SRR7474078_2.fastq \
2 > SRR7474078.firstpass.log | samtools view -hbuS - |samtools sort - \
>SRR7474078.firstpass.bam

samtools view -hf 4 SRR7474078.firstpass.bam \
| samtools view -Sb - > SRR7474078.unmapped.bam

find_circ/./unmapped2anchors.py SRR7474078.unmapped.bam >
SRR7474078.anchors.fastq

bowtie2 \
-p 16 \
--reorder \
--mm \
--score-min=C,-15,0 \
-q \
-x GRCh38 \
-U SRR7474078.anchors.fastq \
2> SRR7474078.secondpass.log \
| find_circ/./find_circ.py \
--genome=GRCh38.primary_assembly.genome.fa \
 -s SRR7474078.stats.log > SRR7474078.bed 2> SRR7474078.reads

# Convert back-splice junction file to SpliceV compatible format
SpliceV/bin/find_circ_convert SRR7474078.bed
```

3. Plot FARSA transcript using SpliceV:

```
SpliceV/bin/SpliceV \
--bam SRR7474078.Aligned.sortedByCoord.out.bam \
-sj SRR7474078.SJ.out.tab.canonical.bed \
-bsj SRR7474078.bed.circles.bed \
-gtf gencode.v29.basic.annotation.gtf \
--gene FARSA \
--filter 3 \
-stranded reverse \
-rnabp RBM3 HNRNPL HNRNPA1 PTBP1 \
-rnabpc red purple blue green \
-fa GRCh38.primary_assembly.genome.fa \
-c 255,140,0
```

Figure 1C

Paired end FASTQ files can be obtained from the NCBI Sequence Read Archive (SRR7474063; RNase R treated, SRR1032145; Poly-A selected)

1. Generate **coverage** and **canonical splice junction** files:

```
STAR \
--genomeDir GRCh38 \
--runThreadN 14 \
--readFilesIn SRR1032145_1.fastq SRR1032145_2.fastq \
--outSAMtype BAM SortedByCoordinate \
--outFileNamePrefix SRR1032145.

# Convert canonical splice junction file to SpliceV compatible format
python SpliceV/bin/star_sj_convert SRR1032145.SJ.out.tab
```

2. Generate **back-splice junction** file with the find_circ pipeline (https://github.com/marvin-jens/find_circ):

```
bowtie2 \
-p 16 \
--very-sensitive \
--phred33 \
--score-min=C,-15,0 \
-x GRCh38 \
--reorder \
--mm \
-q \
-1 SRR7474063_1.fastq \
-2 SRR7474063_2.fastq \
2 > SRR7474063.firstpass.log | samtools view -hbuS - |samtools sort - \
>SRR7474063.firstpass.bam

samtools view -hf 4 SRR7474063.firstpass.bam \
| samtools view -Sb - > SRR7474063.unmapped.bam

find_circ/./unmapped2anchors.py SRR7474063.unmapped.bam >
SRR7474063.anchors.fastq

bowtie2 \
-p 16 \
--reorder \
--mm \
--score-min=C,-15,0 \
-q \
-x GRCh38 \
-U SRR7474063.anchors.fastq \
2> SRR7474063.secondpass.log \
| find_circ/./find_circ.py \
--genome=GRCh38.primary_assembly.genome.fa \
 -s SRR7474063.stats.log > SRR7474063.bed 2> SRR7474063.reads

# Convert back-splice junction file to SpliceV compatible format
SpliceV/bin/find_circ_convert SRR7474063.bed
```

3. Plot GSE transcript using SpliceV:

```
python SpliceV/bin/SpliceV.py \
--bam SRR1032145.Aligned.sortedByCoord.out.bam \
-sj SRR1032145.SJ.out.tab.canonical.bed \
-bsj SRR7474063.bed.circles.bed \
-gtf gencode.v29.basic.annotation.gtf \
--gene GSE1 \
--filter 3 \
-stranded reverse \
-rnabp RBFOX1 MATR3 \
-rnabpc yellow red \
-fa GRCh38.primary_assembly.genome.fa \
-alu alu_elements.bed \
-c blue
```

* alu_elements.bed file obtained from http://genome.ucsc.edu/cgi-bin/hgTables with the following parameters:

group: Repeats
track: RepeatMasker
output format: BED – browser extensible data
output file: rpt.bed

Download the file then run the following command:

```
grep Alu rpt.bed | cut -f1,2,3,6 > alu_elements.bed
```
